# Statistical method on nonrandom clustering with application to somatic mutations in cancer

**DOI:** 10.1186/1471-2105-11-11

**Published:** 2010-01-07

**Authors:** Jingjing Ye, Adam Pavlicek, Elizabeth A Lunney, Paul A Rejto, Chi-Hse Teng

**Affiliations:** 1 Global Pre-Clinical Statistics, Pfizer Global Research and Development, 10777 Science Center Drive, San Diego, CA, 92121, USA; 2 Computational Biology Group, Oncology Research Unit, Pfizer Global Research and Development, San Diego, CA, 92121, USA; 3 Statistics, Corporate Analytics, Amylin Pharmaceuticals Inc, 9360 Towne Centre Drive, San Diego, CA, 92121, USA

## Abstract

**Background:**

Human cancer is caused by the accumulation of tumor-specific mutations in oncogenes and tumor suppressors that confer a selective growth advantage to cells. As a consequence of genomic instability and high levels of proliferation, many passenger mutations that do not contribute to the cancer phenotype arise alongside mutations that drive oncogenesis. While several approaches have been developed to separate driver mutations from passengers, few approaches can specifically identify activating driver mutations in oncogenes, which are more amenable for pharmacological intervention.

**Results:**

We propose a new statistical method for detecting activating mutations in cancer by identifying nonrandom clusters of amino acid mutations in protein sequences. A probability model is derived using order statistics assuming that the location of amino acid mutations on a protein follows a uniform distribution. Our statistical measure is the differences between pair-wise order statistics, which is equivalent to the size of an amino acid mutation cluster, and the probabilities are derived from exact and approximate distributions of the statistical measure. Using data in the Catalog of Somatic Mutations in Cancer (COSMIC) database, we have demonstrated that our method detects well-known clusters of activating mutations in KRAS, BRAF, PI3K, and *β*-catenin. The method can also identify new cancer targets as well as gain-of-function mutations in tumor suppressors.

**Conclusions:**

Our proposed method is useful to discover activating driver mutations in cancer by identifying nonrandom clusters of somatic amino acid mutations in protein sequences.

## Background

Cancer is a genetic disease caused by the accumulation of tumor-specific (somatic) mutations in two broadly defined types of genes called tumor suppressors and oncogenes (Vogelstein and Kinzler (2004) [1]). In general, mutations in tumor suppressors tend to inactivate these natural repressors of tumorgenesis. Mutations in cellular proto-oncogenes, on the other hand, typically increase or deregulate the activity of their protein products. The existence of different types of genes and mutations in cancer has significant practical implications for developing targeted therapies in cancer care. So far, pharmacological restoration of tumor suppression function has been challenging: better success has been achieved by inhibiting activated oncogenes (Weinstein and Joe (2006) [2]). In addition to representing potential therapeutic targets, activating mutations can also be used as biomarkers to identify populations likely to respond to therapies targeting the mutated genes. There is therefore significant interest in identifying those mutations necessary for the cancer phenotype (also known as driver mutations), separating the driver mutations from the random (passenger) mutations that occur as a consequence of the genetic instability typical for human tumors (Cahill et al. (1999) [3]), and furthermore distinguishing activating mutations from inactivating mutations.

Several methods have been developed for the automated prediction of driver oncogenic mutations in individual genes, yet few are suitable for detecting activating mutations. The most straightforward method predicts that driver mutations have a large number of mutations relative to the estimated background mutational rate, after normalizing for gene size (Wang et al. (2002) [4]; see also the "Standard test" in supplementary information for Ding et al. (2008) [5]). Another popular approach predicts that driver mutations have a higher frequency of non-synonymous mutations relative to the background mutation rate (e.g. Bardelli et al. (2003) [6]; Yang et al. (2003) [7]; Samuels et al. (2004) [8]; Davies et al. (2005) [9]; Greenman et al. (2006) [10]; Sjöblom et al. (2006) [11]). These methods are typically used to estimate the total fraction of driver mutations or to detect driver genes, but like predictions based on the phylogenic conservation of protein sequences (see Kaminker et al (2007) [12] and refs. therein), they cannot distinguish between inactivating and activating mutations. In addition, these methods are less suitable to identify driver genes that have low mutation frequencies. Meanwhile, approaches that model the destabilizing effects of mutations on protein structure (see Yip et al. (2006) [13] and refs. therein) are more suitable for tumor suppressor genes. Perhaps the most reliable approach is to classify mutations based on prior knowledge from functional studies, but functional information is by definition not available for novel or poorly characterized genes.

We propose an alternative approach to detect activating mutations in oncogenes, based on the hypothesis that only a small number of specific mutations can activate a protein. To be precise, we hypothesize that a localized cluster of amion acid mutations within a protein sequence, especially in the absence of obvious mutational hotspots, is a fingerprint of selection for the oncogenic phenotype associated with activating driver mutations. Evolutionary studies demonstrate that most amino acids replacements are either neutral or incompatible with protein function (Graur and Li (2000) [14]). Thus, activating mutations should concentrate in a small subset of protein positions and domains, while passenger mutations can be distributed more evenly along the protein sequence reflecting random chance and differences in the mutability of individual DNA codons. Consistent with this hypothesis, activating somatic amino acid mutations cluster in protein kinases (e.g. Bardelli et al. (2003) [6]; Samuels. (2004) [8]; Torkamani and Schork (2008) [15]).

Several methods in the statistics literature can be applied to detect mutation clusters. For example, Naus (1965) [16] proposed a statistical test for the maximum number of points in a fixed length cluster on a line, and developed the probability and expectation. Shortly thereafter, Naus (1966) [17] compared the power of two nonrandom clustering tests on a line; one test is the maximum number of points in a fixed-length non-overlapping interval (e.g. 1 to p, p+1 to 2p, etc. for window length p) and another is the maximum number of points in a fixed-length running interval (e.g. 1 to p, 2 to p+1, etc.), which is also called scan statistics. Scan statistics were further developed and applied by Balakrishnan and Koutras (2002) [18], and Glaz and Zhang (2006) [19] generalized the fixed-length restriction to allow variable-length intervals by determining the maximum of a series of scan statistics each with a fixed window length. While Naus's approach and scan statistics with fixed or variable window lengths are useful, a further generalization to an arbitrary number of points in the interval is more flexible and useful to identify activating driver mutations.

In this work a new statistics method is introduced that identifies nonrandom mutation clustering without specifying the number of mutations or the cluster length. The exact and approximate distribution of the statistical measure is derived and a nonrandom mutation clustering (NMC) algorithm is developed based on the measure. We confirmed the utility of this approach by detecting well-known activating mutations in KRAS, BRAF, PI3K, and *β*-catenin oncogenes, as well as gain-of-function mutations in several tumor suppressors.

## Results

### Data Description

Data used in this study are from COSMIC (Catalog of Somatic Mutations in Cancer) database version 40 (Forbes et al (2008) [20]) via http://www.sanger.ac.uk/cosmic. To ensure compatibility with the test assumption that the location of amino acid mutations on a protein follows a uniform distribution, we limit our analysis to studies annotated as whole gene screens in COSMIC; this eliminated a great majority of COSMIC studies annotated as partial or with missing information on the full-gene screen status. Furthermore, the cluster analysis is restricted to missense mutations: nonsense and synonymous changes are excluded. We limited our search to confirmed somatic variants or mutations that were reported in other studies as somatic. Finally, we removed redundancy in mutations in cancer cell lines, since cell line mutations are often reported by several independent studies.

### Nonrandom clusters in cancer genes

Using the NMC algorithm (see Methods), 12 different proteins out of 446 contain nonrandom amino acid mutation clusters with cutoff probability of less than 0.05, with the most significant clusters listed in Table 1 (probability < 0.01). The clusters include well-known mutation hotspots in classical oncogenes such as BRAF, RAS genes, PI3K, ERBB2/Her2, and CTNNB1/*β*-catenin. Interestingly, nonrandom amino acid mutation clusters were also identified in genes not considered to be classical oncogenes and even a few tumor suppressors. Interpretation of selected positive controls is described below in more detail.

**Table 1:** Genes with significant mutation clusters (Probability < 0.01)

Gene	Cluster size	Cluster positions	Number of mutations in cluster	Cumulative cluster probability*
KRAS (188 aa)	2	12-13	131	1.47E-234

BRAF (766 aa)	1	600-600	60	2.02E-157

TP53 (393 aa)	155	132-286	326	3.07E-101

NRAS (189 aa)	1	61-61	33	7.11E-62

PIK3CA (1068 aa)	5	542-546	27	7.09E-46

CTNNB1 (781 aa)	13	33-45	12	8.54E-19

ERBB2 (1255 aa)	1	776-776	2	7.97e-4

HRAS (189 aa)	1	61-61	4	2.06E-06

PTEN (403 aa)	63	111-173	8	5.50E-05

MAP2K7 (419 aa)	1	162-162	2	0.002386

LRRK2 (2534 aa)	4	1723-1726	2	0.003547

*: only most significant cluster per gene is listed

### Mutation hotspots in classical oncogenes

**Figure 1:**
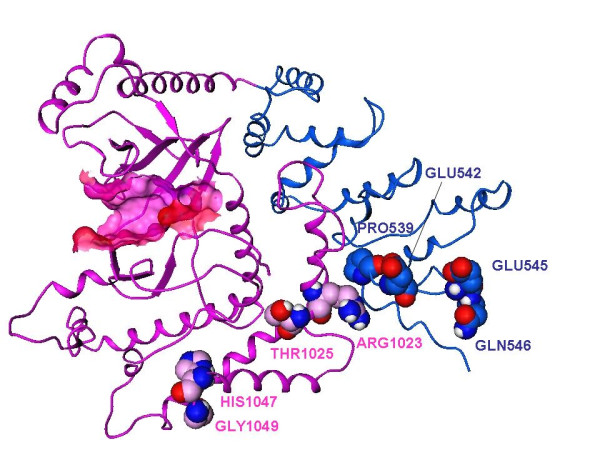
**Ribbon representation of the PI3K*α***. Ribbon representation of the PI3K*α *helical domain (blue) and kinase domain (magenta) extracted from the p110*α*/p85*α *complex (PDB Code: 2RD0; Berman et al. (2000) [45]; Huang et al. (2007) [46]). Displayed in CPK representations are sites of major oncogenic mutations: Pro539, Glu542, Glu545 and Gln546 in the helical domain (blue); Arg1023, Thr1025, His1047 and Gly1049 in the kinase domain (pink). The ATP binding site in the kinase domain is highlighted with a surface.

Table 2 lists the significant clusters obtained from our set of strictly selected COSMIC studies for the BRAF, KRAS, CTNNB1/*β*-catenin, PI3K, and ERBB2 oncogenes. As expected, the most significant hotspot in BRAF was amino acid residue 600 and represents the well-known, highly oncogenic V600E mutation (Davies et al. (2002) [21]). Similarly, RAS residues 12, 13, and 61 are known to be frequently mutated in tumors (see Bos (1988) [22] for review). Residues 33, 34, 37, 41, and 45 were identified as a significant cluster in CTNNB1/*β*-catenin. There is a clear mechanistic rationale: residues 33, 37 and 41 are phosphorylated directly by GSK-3 *β *while residue 45 has been reported to be a primer site that is phosphorylated by CK1 (Hagen and Vidal-Puig (2002) [23]): mutations at these positions prevent GSK-3 *β*-mediated degradation of *β*-catenin (Morin et al. (1997) [24]). Residues 542-546 surround the 545 hotspot in the helical domain of PI3K/PIK3CA (Samuels et al. (2004) [8]), with a second significant PI3K cluster in the kinase domain (positions 1025-1049; probability 2.60E-20; Figure 1). Mutation of Glycine 766 in ERBB2 has been reported to dramatically increase protein kinase activity (Fan et al. (2008) [25]).

**Table 2:** Mutation positions for selected oncogenes

Gene	Position (#of mutations)
BRAF(766 aa)	464(1), 466(2), 469(4), 581(1), 596(2), 597(2), **600**(60), 601(2)

KRAS(188 aa)	**12**(99), **13**(32), 22(1), 23(1), 61(6), 117(1), 146(10)

CTNNB1 (781 aa)	6(1), **33**(3), **34**(2), **37**(3), **41**(2), **45**(2)

PIK3CA(1068 aa)	88(3), 111(3), 118(1), 124(1), 345(1), 449(1), 453(1), 539(1), **542**(5), **545**(20), **546**(2), 549(1), 1023(1), 1025(1), 1047(21), 1049(1), 1066(1)

The number of mutations for each position is shown in parenthesis, positions within clusters from Table 1 are highlighted in bold, and CpG positions are underlined.

For most genes in Table 1, multiple significant amino acid clusters were found, with the most significant clusters in sites of well-known oncogenic mutations. Interestingly, the great majority of the mutation-hotspots are not in CpG positions (Table 2) suggesting that selection and not the underlying mutation rate drives these changes in tumors.

### General remarks on detected mutation hotspots

In addition to known clusters of activating mutations in major oncogenes, several other genes have significant mutation hot-spots. For example, two mutations between the Roc (Ras of complex proteins) and kinase domains in the LRRK2 locus form a significant cluster. The LRRK2 kinase, also known as PARK8, is not considered to be a classical cancer gene. It most closely resembles the family of tyrosine-like kinases that phosphorylate serine/threonine residues and lies upstream of mitogen-activated protein kinase (MAPK) pathways (Mata et al. (2006) [26]). Interestingly, germline polymorphisms in LRRK2 predispose affected individuals to Parkinson disease and are linked to specific cancer types (Inzelberg and Jankovic (2007) [27]; Strongosky et al. (2008) [28]).

**Figure 2:**
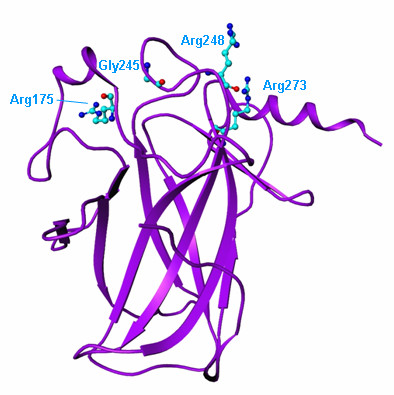
**Ribbon representation of the human p53**. Ribbon representation of the human p53 core domain X-ray structure (PDB Code: 2OCJ; Wang et al. (2007) [47]). Displayed in CPK representation are sites of major oncogenic mutations: Arg175, Gly245, Arg248 and Arg273.

As expected, we found fewer significant mutation hot-spots in tumor suppressors, and these hot-spots were typically much larger than those associated with oncogenes. In general, inactivating amino acid mutations are not expected to form localized nonrandom clusters, but rather to span many residues in highly conserved regions (e.g. Nigro et al. (1989) [29]). The most significant cluster identified in TP53/p53 spans residues 132-286, one of the four major p53 mutation hotspots that are highly conserved in vertebrates (Nigro et al. (1989) [29]). It overlaps the original major hotspot in residues 110-307 identified by Hollstein et al. (1991) [30], and spans two shorter hotspots that include gain-of-function mutations in positions 248 and 273 (Song et al. (2007) [31]). Structural analysis demonstrates that both regions are close together in the folded protein (Figure 2). The cluster found in the phosphatase and tensin-homology domains of PTEN includes residues known to inhibit PTEN phosphatase activity (Tolkacheva and Chan (2000) [32]), and sequence conservation cannot explain this clustering since most of the PTEN protein is well conserved among vertebrates (Yu et al. (2001) [33]). These examples demonstrate that tumor suppressor activity can be muted by changes in protein function in addition to gene deletions or disruption of the reading frame.

## Discussion and Conclusions

A new method for the identification of nonrandom mutation clusters in biological sequences is presented. The method is fast, robust, and unlike many previous methods, it is does not require a fixed window length, which enables the identification of significant clusters of variable sizes, particularly important for the detection of activating mutations. We have applied this method to investigate somatic amino acid mutations in the COSMIC database. Our method detected very short clusters spanning a few individual amino acid positions in the case of the oncogenes BRAF or KRAS, as well as larger regions in the tumor suppressors p53 and PTEN.

A recent paper by Wagner (2007) [34] proposed two similar approaches using the distance between mutation positions. In the first approach, a Poisson distribution was utilized to model mutation clusters. The test on the distance of mutation positions containing *k *mutations was derived and the minimum *k *that gives significance was determined. The second approach assumed a uniform hypothesis and used permutation testing for significance. The permutation test is an approximation whose precision depends on the number of permutations undertaken, which can be very computationally intensive for good accuracy and precision. By comparison, our measure on distance is based on a uniform distribution and is calculated directly via order statistics.

Our method has several potential limitations. First of all, the status of all coding positions must be determined. This is primarily a limitation for older studies, where typically only those exons with known mutations were screened. However, with the explosion of large-scale cancer genome sequencing (e.g. Sjöblom et al. (2006) [11]; Greenman et al (2007) [10]; Jones et al. (2008) [35]; Parsons et al. (2008) [36]; Cancer Genome Atlas Research Network. (2008) [37]), the number of studies suitable for analysis by NMC will grow. Another limitation is our assumption that the mutation probability is uniform: hypermutable positions for both germline and somatic mutations have been reported. As a result, we have excluded all insertions and deletions, since these mutations have strong sequence-dependence, and restricted analysis to single-point amino acid substitutions. Examples of single point mutation hotspots are CpG dinucleotides, which in unselected genomic sequences have more than ten-fold higher mutation frequency compared to other dinucleotides (Sved and Bird (1990) [38]). CpG hypermutability has been also reported in certain tumors (Jones et al. (1992) [39]). However, as shown in Table 2, only a few of the activating mutations identified by the NMC algorithm are in CpG sites. Another potential bias can be introduced by an unequal rate of mutagenesis caused by deficient repair of DNA damage in cells and environmental mutagens. For instance, tobacco smoke preferentially induces G to T transversions in DNA in lung cancer while colorectal tumors exhibit more transitions than transversions (Hollstein et al. (1991) [30]). Yet, despite the fact that lung and colorectal tumors have different mutational spectra, essentially all KRAS mutations in these tumors occur in residues 12, 13, and 61. In summary, while our analysis is affected by nonrandom factors such as the presence of mutation hotspots or exposure to different mutagens, positive selection for a cancer phenotype appears to be the major cause of mutation clustering.

The aim of the method is to detect activating mutations that are assumed to be concentrated in specific amino acid positions. Activating mutations are typical for cellular proto-oncogenes and, as expected, significant clusters are detected in oncogenes such as BRAF, RAS genes, CTNNB1/*β*-catenin, or PI3K. Less intuitive, however, are positively selected residues in the p53 and PTEN tumor suppressors. Previous reports revealed that these genes encode functional domains that can result in gain-of-(non-suppressor)-function when altered by mutation. Thus, our method may also identify positive selection on mutations that alter the repressive function of tumor suppressors.

In conclusion, we propose a new method for discovering nonrandom clusters of mutations in biological sequences. Unlike previous approaches, the method does not use fixed length windows and therefore can be used to detect clusters of highly variable sizes. We demonstrated the value of this method to detect activating amino acid mutations in human tumors and confirmed nonrandom clustering of well-known oncogenic mutations in several classical oncogenes. The method can be also used to discover new oncogenes from large-scale cancer genome data and to identify gain-of-function mutations in tumor suppressors. Finally, detection of nonrandom sequence changes is a general problem and the method may be useful in other areas such as DNA polymorphism analysis and comparative evolutionary studies (Wagner (2007) [34]).

## Methods

Single amino acid mutations may lead to changes in protein function. Because missense mutations are the most likely single-point genetic mutation to have an effect on protein function, the nonrandom mutation clustering (NMC) algorithm is applied to missense mutations in individual genes in this work.

The NMC algorithm is derived under the following assumptions: 1. each amino acid residue in a protein sequence has equal mutation probability; 2. mutations between amino acid positions are independent; 3. mutations between samples are independent; and 4. the number of potentially available samples is larger than the number of mutations.

Denote *N *as the protein sequence length and *n *as the total number of mutations in the protein. Denote *X*_*i*_, a random variable between 1 and *N*, to be the position of the ith non-synonymous (missense) mutation. By assumption, the mutations follow a discrete uniform distribution, and the *n *mutations are equivalent to *n *independent sample draws with replacement from the discrete uniform distribution, where the probability Pr(*X*_*i *_= j) = 1/N, where j = 1,..., *N *and i = 1,..., *n*.

By assumption, mutations are random and can occur at the same position more than once. The data are transferred into order statistics by ordering the *X*_*i *_into *X*_(1) _≤...≤ *X*_(*i*) _≤...≤ *X*_(*n*)_, where *X*_(*i*) _is the ith smallest number in the sample, i = 1,..., *n*. To characterize clustering, the distance between order statistics *R*_*ki*_= *X*_(*k*) _- *X*_(*i*)_, for any pair i, k, i < k, i, k = 1, .., *n *is computed. We develop the distribution of *R*_*ki*_, and declare the clustering to be nonrandom when the probability that the distance between order statistics *R*_*ki *_is less than a pre-defined significant probability level *α*: Pr(*R*_*ki *_≤ *r*) ≤ *α*. The probability Pr(*R*_*ki *_≤ *r*) is the cumulative distribution of *R*_*ki*_, the chance that the distance between order statistics *X*_(*i*) _and *X*_(*k*) _is as close or closer than *r*. Therefore, the probability Pr(*R*_*ki *_≤ *r*) is derived as a p-value, where the probability *α *is an arbitrary level such as 0.01, 0.05, or 0.1. The distance *R*_*ki *_has the simple interpretation of the size of the mutation cluster.

### 1.1 Derivation of the distribution of statistical measure

While distributions of order statistics are usually derived for continuous distributions, they have also been derived for discrete distributions. Burr (1955) [40] derived the distribution of range statistics using order statistics on a discrete uniform distribution. Range statistics is a special case of our statistical measure *R*_*ki*_, where i = 1 and k = *n*. Evans et al. (2006) [41] developed the density function and cumulative distribution of the ith order statistics given an arbitrary discrete distribution, i = 1,..., *n*. We extend the approach of Evans et al. (2006) [41] to determine the distribution of the distance between order statistics, and generalize the approach of Burr, I.W. (1955) [40] to derive the distribution of statistics *R*_*ki*_.

The distribution of *R*_*ki *_is developed from the joint distribution of order statistics *X*_(*i*) _and *X*_(*k*) _for any pair i, k, i < k, i, k = 1, .., *n*. *R*_*ki*_, the distance between order statistics *X*_(*i*) _and *X*_(*k*)_, can range from 0, which means both mutations are located at the same position, to *N*-1, which means the mutations are on the first and last positions of the protein sequence. Intermediate values between 0 and *N*-1 are also possible, for example *R*_*ki *_= 1 implies that the mutations are adjacent to each other and so on. We develop the distribution of *R*_*ki *_for each possible scenario.

*R*_*ki *_= 0, for any pair i, k, i < k, i, k = 1, .., *n*, implies that mutations *X*_(*i*) _and *X*_(*k*) _are located at the same position. Taking the *N *possible positions into consideration, the probability that *R*_*ki *_= 0 is written as

The distribution is derived using the properties of order statistics. For example, when *y = X*_(*i*) _= *X*_(*k*) _= 1, the first *k *order statistics are on the first position and the remaining *n-k *order statistics are on or above the first position. Among these *n-k *order statistics, *v *order statistics are located strictly above the first position, with the remaining *n-k-v *order statistics at the first position, where *v *can range from 0, meaning all *n *order statistics are on the first position, to *n-k*, indicating that all the remaining order statistics are strictly larger than the first position. A similar logic applies to *y *= *X*_(*i*) _= *X*_(*k*) _= *N*. For1 <*y *<*N*, the distribution is derived as follows: there must be *i*-1 order statistics at position *x*, where *x *≤ *y *; among those *i*-1 order statistics, there are *u *order statistics where *x *<*y *and *i-1-u *with *x *= *y*, where *u *can range from 0 to *i*-1. There must be *k-i*+1 order statistics at position *x *= *y*. Finally, there must be *n-k *order statistics at *x*, where *x *≥ *y*; among those *n-k *order statistics, there are *v *order statistics where *x *>*y *and *n-k-v *where *x *= *y*, where *v *can range from 0 to *n-k*. Putting all the terms together, there are *u *order statistics located before position *y*, with probability (*y *- 1) *N*, where *u *= 0,..., *i*-1; there are *(k-i*+1)+(*i*-1-*u*)+(*n*-*k*-*v*) = *n*-*u*-*v *order statistics at *y *with probability1/*N *; there are *v *order statistics after position *y*, with probability1 - *y/N*, where *v *= 0,..., *n-k *and *x *= 2,..., *N*-1. 

For *R*_*ki *_= 1, for any pair i, k, i < k, i, k = 1, .., n, the order statistics *X*_(*i*) _and *X*_(*k*) _are adjacent to each other. The probability distribution can be written as:

For *R*_*ki *_= *r*, for any pair i, k, i < k, i, k = 1, .., *n*, *r *= 2,..., *N*-1, the distribution can be written as:

The distributions for *R*_*ki *_= 1 and *R*_*ki *_= *r *derived above, for any pair i, k, i < k, i, k = 1, .., *n*, *r *= 2,..., *N*-1, is based on similar logic as *R*_*ki*_= 0. The *i*-1 order statistics must be located at or before position *X*_(*i*)_, and the *n-k *order statistics must be located at or after position *X*_(*k*)_. For the remaining *k-i*-1 order statistics, *q *order statistics are located at position *X*_(*i*)_, *t *order statistics are strictly between *X*_(*i*) _and *X*_(*k*) _and the remaining *k-i*-1*-q-t *statistics are at position *X*_(*k*)_, where *q *= 0,..., *k-i*-1 and *t *= 0,..., *k-i-*1*-q*. Grouping all the terms together yields the distribution equations for *R*_*ki *_= 1 and *R*_*ki *_= *r*, for any pairs of i, k, i < k, i, k = 1, .., *n*, *r *= 2,..., *N*-1.

Finally, for the special case of i = 1 and k = *n*, the distribution of *R*_*ki *_may be simplified as

Note that Pr(R_*n*1 _≤ *r*) = 1 for *r *= *N*-1. The result is the same as the range statistics reported in Burr, I.W. (1955) [40].

### 1.2 Approximation of the distribution

The derivation in section 1.1 is the exact distribution of the statistical measure for nonrandom mutation clustering in the discrete uniform distribution. Proteins typically contain hundreds or thousands of amino acids and it is convenient to approximate the discrete uniform distribution with a continuous uniform distribution (0, 1) because calculating the distribution of *R*_*ki *_= *r *can be extremely slow when the length of the protein sequence *N *or the number of mutations *n *is large, resulting in dramatically increased iterations in those summations. For computational efficiency, we now develop the distribution for the test statistics in the continuous limit.

When the *n *order statistics are random samples from a uniform distribution (0, 1), the probability distribution of order statistics *X*_(*i*) _and *X*_(*k*)_, for any pair i, k, i < k, i, k = 1, .., is:

where distance is normalized to be in the range (0,1), so the distance *R*_*ki *_= (*X*_(*k*) _- *X*_(*i*)_)/*N *differs by the constant *N *from section 1.1, where *R*_*ki*_= *X*_(*k*) _- *X*_(*i*)_. The cumulative distribution can be written as Pr(*R*_*ki *_≤ *r*)

which by iterated integration by parts gives:

Using the continuous uniform distribution, *R*_*ki *_simply follows a Beta distribution with parameters *k-i *and *i *+ *n *- *k *+ 1, ensuring that Pr(*R*_*ki *_≤ 1) = 1. This result was reported in Johnson et al. (1995) [42] for a joint distribution of pair-wise order statistics following a continuous uniform distribution (0, 1).

### 1.3 Correction for multiple testing

For each pair-wise order statistic, the exact and continuous distributions can be calculated using formulas in sections 1.1 and 1.2. Clusters are evaluated for each pair of order statistics, which can elevate the false positive rate due to multiple testing. A Bonferroni correction can be chosen to correct the false positive rate because it doesn't require an independent hypotheses assumption and it is a conservative test. The false discovery rate (FDR) developed by Benjamini and Hochberg (1995) [43] is popular and has been applied to multiple testing problems in many areas. Although it requires an independent test statistics assumption, it is known to be powerful and robust under positively correlated test statistics (Benjamini and Yekutieli (2001) [44]). Because of its conservativeness, Bonferroni is applied as the default to adjust multiple testing for the NMC algorithm and as an alternative, FDR can be applied.

### 1.4 NMC algorithm

The exact and approximate distributions of distance between pair-wise order statistics were derived in section 1.1 and 1.2. The calculation is rapid for the special case when *R*_*ki *_is 0 or 1 or for the range statistics, and we use the exact distribution derived in section 1.1 to ensure accuracy for these cases. For further efficiency when calculating the distribution for *R*_*ki *_= 1, the algorithm is stopped when the iterated summation in the distribution reaches the significance level because the full summation is larger than the partial summation and the difference cannot be significant. The continuous distribution is used for computational efficacy when the difference *R*_*ki *_is greater than 1. The nonrandom mutation clustering (NMC) algorithm is summarized in the following procedure:

• **Input**: Number and location of missense mutations in a protein

• **Output**: A table with columns of nonrandom mutation cluster size, starting location of the cluster, ending location of the cluster, number of mutations observed in the cluster and probability of the cluster that is significant after Bonferroni or FDR correction.

• **NMC algorithm**:

◦ Step 1: Reorder the mutation positions into order statistics and set the significance level *α*. By default, *α *= 0.05.

◦ Step 2: For each pair-wise order statistics, calculate the probability Pr(*R*_*ki *_≤ *r*), for any pair i, k, i < k, i, k = 1, .., *n*. For R = 0 and 1 and/or i = 1 and k = *n*, use the distribution in section 1.1. For r>1, use the distribution in section 1.2.

◦ Step 3: Calculate the Bonferroni or FDR corrected probabilities.

◦ Step 4: Report the multiple-testing corrected significant clusters in the output table after sorting from the lowest probability to the highest.

The R source code is available in Additional file 1 and an analysis of minimum number of mutations required for NMC algorithm is available in Additional file 2.

## Competing interests

The authors declare that they have no competing interests.

## Authors' contributions

JY designed and developed the statistical method, and coded the NMC algorithm in R. AP and PAR proposed the idea of detecting activating mutations with nonrandom clusters. AP acquired the COSMIC database and prepared the data. JY and AP performed the analysis and drafted the manuscript. EAL and PAR contributed the idea of three-dimensional mutation detection. CT contributed the idea of the statistical method. EAL, PAR and CT revised the manuscript. PAR finalized the manuscript. All authors read and approved the final manuscript.

## Supplementary Material

Additional file 1 **NMC**. R source code of NMC algorithm. Click here for file

Additional file 2 **Poweranalysis**. Analysis of minimum number of mutations required for NMC algorithm Click here for file
