# Supplementary material for: Statistical method on nonrandom clustering with application to somatic mutations in cancer
Source: BMC Bioinformatics. 2010 Jan 7;11:11. doi: 10.1186/1471-2105-11-11 (PMC2822753; doi:10.1186/1471-2105-11-11)
Supplement: Additional file 1 — NMC. R source code of NMC algorithm. [file 1471-2105-11-11-S1.DOC]

######################################################

#NMC.r is a collection of R functions to detect nonrandom mutation clustering (NMC) on gene sequences

#Copyright (C) December, 2008 created by Jingjing Ye (Jingjing.Ye@pfizer.com)

#This code is the first version of NMC; this code is released under

#the GLP license version 2(http://www.gnu.org/copyleft/gpl.html)

#This program is free software; you can redistribute it and/or

#modify it under the terms of the GNU General Public License

#as published by the Free Software Foundation; either version 2

#of the License, or (at your option) any later version.

#This program is distributed in the hope that it will be useful,

#but WITHOUT ANY WARRANTY; without even the implied warranty of

#MERCHANTABILITY or FITNESS FOR A PARTICULAR PURPOSE. See the

#GNU General Public License for more details.

#You should have received a copy of the GNU General Public License

#along with this program; if not, write to the Free Software

#Foundation, Inc., 51 Franklin Street, Fifth Floor, Boston, MA 02110-1301, USA.

#######################################################

#range statistics

#calculate distrbution of range for discrete uniform distribution

#n is the number of mutation, N is the length of gene sequence

rangestat <- function(n,N){

rangescdf <- matrix(0,1,N)

rangespdf <- matrix(0,1,N)

rangescdf[,1] <- rangespdf[,1] <- (1/N)^(n-1)

for(i in 1:(N-1)){

rangescdf[,(i+1)] <- rangescdf[,i] + (N-i)*(((i+1)/N)^n-2*(i/N)^n+((i-1)/N)^n)

rangespdf[,(i+1)] <- (N-i)*(((i+1)/N)^n-2*(i/N)^n+((i-1)/N)^n)

}

colnames(rangescdf) <- 0:(N-1)

colnames(rangespdf) <- 0:(N-1)

ranges <- list(rangescdf,rangespdf)

names(ranges) <- c("cdf","pdf")

ranges

}

#Function for calculation when mutations on same location (r=0)

#N is the sequence length, n is the number of mutations

#i is the ith order statistical and j is the jth order statistics, i<j, i,j=1,...,n

difforder0 <- function(N,n,i,j){

diffs <- matrix(0,1,N)

diffs[,1] <- 1-pbinom(j-1,n,prob=1/N)

diffs[,N] <- pbinom(i-1,n,prob=(N-1)/N)

for(x in 2:(N-1)){

Mp <- c((x-1)/N,1/N,1-x/N)

for(w in 0:(i-1)){

for(u in 0:(n-j)){

mr <- c(w,n-w-u,u)

diffs[,x] <- diffs[,x] + dmultinom(mr,prob=Mp)

}

}

}

diffs

}

#Function for calculation when mutations are next to each other (r=1)

#N is the sequence length, n is the number of mutations

#i is the ith order statistical and j is the jth order statistics, i<j, i,j=1,...,n

#r is the difference between ith and jth order statistics

difforder1 <- function(N,n,i,j,r=1){

diffs <- matrix(0,1,N-r)

Mp <- c(1/N,1/N,1-2/N)

for(u in 0:(n-j)){

for(w in 0:(j-i-1)){

mr <- c(i+w,j-i-w+u,n-j-u)

diffs[,1] <- dmultinom(mr,prob=Mp)+ diffs[,1]

}

}

if(N-r > 1){

for(x in 2:(N-r)){

Mp <- c((x-1)/N,1/N,1/N,1-(x+1)/N)

for(u in 0:(i-1)){

for(w in 0:(j-i-1)){

for(qs in 0:(n-j)){

mr <- c(i-1-u,u+1+w,j-i-w+qs,n-j-qs)

diffs[,x] <- diffs[,x] + dmultinom(mr,prob=Mp)

}

}

}

}

}

diffs

}

###################################################

#main function to run NMC; function will call upon previous functions

#nmc is NMC algorithm

#Input:

#x is matrix of mutation data with 0 as wild type and 1 as mutation

#alpha is significance level

#mulltest is p-value is adjusted by Bonferroni method, Benjamini-Hochberg or not; by default, it is Bonferroni

#Output:

#A result matrix with five columns:

#cluster size, starting location of the cluster, ending location of the cluster, number of mutations in the cluster, p-value of the cluster

#P-values are reported from lowest p-value to the highest

#only p-values less than alpha (or Bonferroni/BH corrected alpha) are listed

##################################################

nmc <- function(x,alpha=0.05,multtest=c("Bonferroni","BH","None")){

library(multtest)

N <- dim(x)[2]

n <- length(which(x==1))

newx <- apply(x,2,sum)

mp <- which(newx != 0)

mp2 <- cumsum(newx[mp])

pvalues2 <- matrix(0,length(mp),2) #same position

temp <- difforder0(N,sum(newx),1,mp2[1])

pvalues2[1,1] <- mp2[1]

pvalues2[1,2] <- sum(temp)

if(length(mp) > 1){

for(i in 2:length(mp)){

if((mp2[i-1]+1) != mp2[i]){

temp <- difforder0(N,sum(newx),(mp2[i-1]+1),mp2[i]) }

else {

temp <- 1 #if one position has one number

}

pvalues2[i,1] <- newx[mp[i]]

pvalues2[i,2] <- sum(temp)

}

}

pvalues2 <- cbind(mp,mp,pvalues2)

multtest <- match.arg(multtest)

#Bonferroni correction

if(multtest == "Bonferroni"){

criterion <- alpha/(length(mp) + (length(mp)*(length(mp)-1)/2))

}

else{

criterion <- alpha

}

if(n > 1){

if(length(mp) > 1){

pvalues <- matrix(0,(length(mp)*(length(mp)-1)/2),2)

tmp <- rep(mp[1],length(mp)-1)

tmp2 <- mp[-1]

tmp3 <- rep(1,length(mp)-1)

tmp4 <- mp2[-1]

for(i in 2:length(mp)){

tmp <- c(tmp,rep(mp[i],length(mp)-i))

tmp2 <- c(tmp2,mp[-(1:i)])

tmp3 <- c(tmp3,rep(mp2[i-1]+1,length(mp)-i))

tmp4 <- c(tmp4,mp2[-(1:i)])

}

for(i in 1:dim(pvalues)[1]){

if(((tmp2[i]-tmp[i]) == (max(tmp2)-min(tmp)))){

temp <- rangestat(sum(newx),N)

temp <- temp$cdf[(max(tmp2)-min(tmp))+1]

pvalues[i,1] <- sum(newx[tmp[i]:tmp2[i]])

pvalues[i,2] <- sum(temp)

}

else if((tmp2[i]-tmp[i]) == 1){

temp <- difforder1(N,sum(newx),tmp3[i],tmp4[i])

if((sum(temp) != 0) & (sum(temp) <= criterion)){

temp2 <- difforder0(N,sum(newx),tmp3[i],tmp4[i])

temp <- c(temp,temp2)

}

pvalues[i,1] <- sum(newx[tmp[i]:tmp2[i]])

pvalues[i,2] <- sum(temp)

}

else{

pvalues[i,1] <- sum(newx[tmp[i]:tmp2[i]])

pvalues[i,2] <- pbeta(q=abs(tmp[i]-tmp2[i])/N, shape1=tmp4[i]-tmp3[i], shape2=n-tmp4[i]+tmp3[i]+1)

}

}

pvalues <- cbind(tmp,tmp2,pvalues)

pvalues <- rbind(pvalues,pvalues2)

pvalues <- pvalues[order(pvalues[,4]),]

pvalues <- cbind((pvalues[,2]-pvalues[,1]+1),pvalues)

colnames(pvalues) <- c("cluster_size","start","end","number","p_value")

if(multtest == "BH"){

ap <- mt.rawp2adjp(pvalues[,5],"BH")

pvalues[,5] <- ap$adjp[order(ap$index),2]

if(length(which(as.numeric(pvalues[,5]) <= criterion)) >= 1){

pvalues <- pvalues[which(as.numeric(pvalues[,5]) <= criterion),]

}

else {

pvalues <- NULL

}

pvalues

}

else{

if(length(which(as.numeric(pvalues[,5]) <= criterion)) >= 1){

pvalues <- pvalues[which(as.numeric(pvalues[,5]) <= criterion),]

}

else {

pvalues <- NULL

}

pvalues

}

}

else{

pvalues <- cbind((pvalues2[,2]-pvalues2[,1]+1),pvalues2)

colnames(pvalues) <- c("cluster_size","start","end","number","p_value")

if(multtest == "BH"){

ap <- mt.rawp2adjp(pvalues[,5],"BH")

pvalues[,5] <- ap$adjp[order(ap$index),2]

if(length(which(as.numeric(pvalues[,5]) <= criterion)) >= 1){

pvalues <- pvalues[which(as.numeric(pvalues[,5]) <= criterion),]

}

else {

pvalues <- NULL

}

pvalues

}

else{

if(length(which(as.numeric(pvalues[,5]) <= criterion)) >= 1){

pvalues <- pvalues[which(as.numeric(pvalues[,5]) <= criterion),]

}

else {

pvalues <- NULL

}

pvalues

}

}

}

else{

pvalues <- cbind((pvalues2[,2]-pvalues2[,1]+1),pvalues2)

colnames(pvalues) <- c("cluster_size","start","end","number","p_value")

if(multtest == "BH"){

ap <- mt.rawp2adjp(pvalues[,5],"BH")

pvalues[,5] <- ap$adjp[order(ap$index),2]

if(length(which(as.numeric(pvalues[,5]) <= criterion)) >= 1){

pvalues <- pvalues[which(as.numeric(pvalues[,5]) <= criterion),]

}

else {

pvalues <- NULL

}

pvalues

}

else{

if(length(which(as.numeric(pvalues[,5]) <= criterion)) >= 1){

pvalues <- pvalues[which(as.numeric(pvalues[,5]) <= criterion),]

}

else {

pvalues <- NULL

}

pvalues

}

}

}
