# Supplementary material for: Statistical method on nonrandom clustering with application to somatic mutations in cancer
Source: BMC Bioinformatics. 2010 Jan 7;11:11. doi: 10.1186/1471-2105-11-11 (PMC2822753; doi:10.1186/1471-2105-11-11)

Power analysis of NMC algorithm

The minimum number of mutations needed for our method is 2. Note that this number is independent of the frequency of the mutations in any sample, and the method is therefore applicable to detect activating mutations that occur at arbitrarily low frequency. We derived the results from theoretical results using range statistics, reported in the Methods section of the paper. The following plot shows how close the mutations must be in order to be called significant at 0.05 level for different sequence length: 100, 200, 500, 1000, 2000 and 3000. From the plot, when the sequence length is 100, a cluster with two mutations is significant if the mutations are closer than two positions on the sequence; for sequence length 200, a cluster is significant if the mutations are closer than four positions, etc.


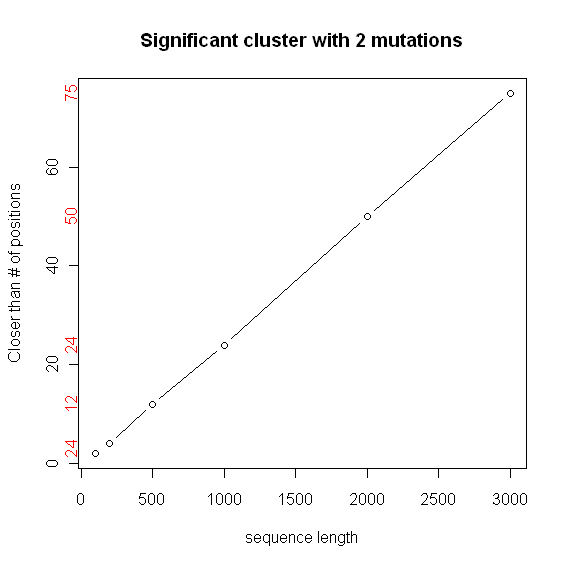

Supplement: Additional file 2 — Poweranalysis. Analysis of minimum number of mutations required for NMC algorithm [file 1471-2105-11-11-S2.DOC]
